# Released Volatile Organic Compounds in Southern Yellow Pine before and after Heat Treatment

**DOI:** 10.3390/ijerph15112579

**Published:** 2018-11-18

**Authors:** Chen Wang, Zhiping Wang, Ye Qin, Xiaoqian Yin, Anmin Huang

**Affiliations:** Research Institute of Wood Industry, Chinese Academy of Forestry, Beijing 100091, China; wonderfulmorning@163.com (C.W.); 15321593606@163.com (Z.W.); qinye@caf.ac.cn (Y.Q.); yxqwzy@163.com (X.Y.)

**Keywords:** heat treatment, southern yellow pine, volatile organic compounds

## Abstract

As the main material in indoor furniture, southern yellow pine (*Pinus* spp.) releases volatile organic compounds (VOCs) into the environment during use. To better understand variations in the contents of VOCs in southern yellow pine before and after heat treatment, this study conducts dry heat treatment on southern yellow pine at 140 °C and 220 °C. Headspace solid phase micro-extraction was used to extract VOCs from southern yellow pine. The VOCs of southern yellow pine before and after heat treatment were identified via gas chromatography-mass spectrometry, and chemical component differences were characterized via Fourier transform infrared spectroscopy. Results reveal 86 VOCs in pure southern yellow pine, including alcohols, aromatics, acids, aldehydes, alkanes, alkenes, and some trace compounds (e.g., furans, ketones, phenols, and esters). With an increase in heat-treatment temperature, the contents of alkanes increased, whereas those of alcohols and alkenes decreased. The contents of aromatics, acids, and aldehydes were highest when heat treated at 140 °C. At 220 °C, the total contents of key VOCs in southern yellow pine were lowest.

## 1. Introduction

Southern yellow pine (*Pinus* spp.) is a type of softwood with a beautiful natural texture and appearance. It has relatively high strength among softwood species. Because of its excellent structural performance, southern yellow pine is widely used in structural construction under various conditions, such as outdoor landscape facilities, flooring materials, and in children’s beds. However, due to the high content of pine oil and extract in southern yellow pine, it can easily release volatile organic compounds (VOCs) into the environment. Such VOCs include alcohols, aldehydes, ketones, esters, ethers, aromatic hydrocarbons, terpenes, amines, and other substances [1]. Most VOCs in natural wood are harmless to humans, but excessive VOCs can cause irritation to the human body due to odor [2]. Such odors are often thought to be due to formaldehyde; however, a lack of understanding of odor sources and potential threats to human health has caused conflict between consumers and furniture enterprises. Studies have shown that VOCs originate from wood extracts [3]. Liu et al. compared VOC contents in Cathy poplar, rubber wood, southern yellow pine, and China fir before and after extraction for 6 h and determined that VOC contents in extracted wood declined substantially [4,5]. However, because of the high-cost, it is difficult to extract wood on a large scale in wood industry applications. Heat treatment is an effective wood processing technique and can improve hydrophobic properties, dimensional stability, and fungal resistance without using wood preservatives [6,7,8,9]. However, heat treatment can also reduce the extractive content in wood. An increase in heat-treatment temperature can volatilize or degrade a large amount of original extractives [10]. Heat treatment can also change VOC contents in southern yellow pine, but there have been few studies in this area.

Gas chromatography-mass spectrometry (GC-MS) offers clear advantages when analyzing complex mixtures; by combining an ideal separation technique (GC) with a sensitive identification approach (MS), GC-MS constitutes a reliable and common method for qualitative and quantitative compound analyses [11,12]. Because of high detection sensitivity and good separation effect, GC-MS has been widely applied in identifying VOCs in perfume [13], food [14,15], and textiles [16,17]. VOC characterization and identification in wood have also been well documented [18].

To better understand variations in VOC contents in southern yellow pine before and after heat treatment, this paper uses GC-MS to identify VOC changes and employs Fourier transform infrared spectroscopy (FTIR) to analyze chemical component differences in untreated wood and heat-treated wood, which can provide a basis for wood heat-treatment processes and applications. This paper can also help consumers better understand VOCs released from wood products.

## 2. Materials and Methods

### 2.1. Materials

The logs were purchased from Shanghai Aiwei Industrial Development Co., Ltd. (Shanghai, China); details are listed in Table 1. The logs were obtained from mature wood above breast height without any cracking, decay, discoloration, or other defects.

### 2.2. Preparation of Wood Samples

The logs were processed into experimental samples which size are 10 × 10 × 10 mm. Then the experimental samples were placed in an air-drying oven (Shanghai Bosun Medical Biological Instrument Co, Ltd., Shanghai, China) at 103 °C for 8 h until the samples were completely dry.

### 2.3. Heat Treatment

**Figure 1:**
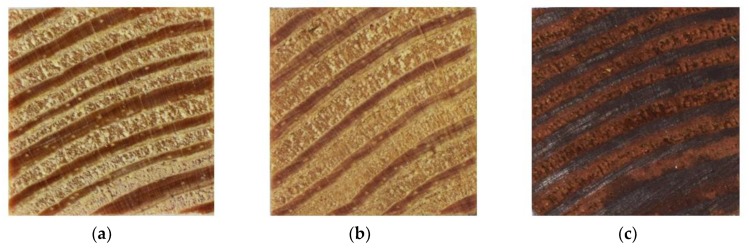
Images of partial heat-treated wood samples. (**a**) Untreated southern yellow pine; (**b**) heat treated at 140 °C; (**c**) heat treated at 220 °C.

Seventy-five experimental samples were divided into three groups equally. The first group (with no treatment) was the control; the second and third groups were placed into an air-drying oven (Shanghai Bosun Medical Biological Instrument Co., Ltd., Shanghai, China) for 6 h at respective temperatures of 140 °C and 220 °C. Images of partial heat-treated wood samples are presented in Figure 1.

After heat treatment, the wood samples were ground into fibers of 40–60 mesh consistent with approximate particle sizes of 50 μm (Deqing Baijie Electric Appliance Co., Ltd., Deqing, China).

### 2.4. Fourier Transform Infrared Spectroscopy

FTIR analyses were carried out at room temperature according to the methodology described by NICOLET 6700, using a Spectrum 2000 FTIR (Thermo Fisher Scientific, Waltham, MA, USA) with a universal attenuated total reflectance (UATR) accessory. Potassium bromide (KBr) was used to collect background spectra. Air-dried sample powders were mixed with KBr at a weight ratio of 1:100 before spectrum collection. Ten scans were carried out for each sample in a spectral range from 400 to 4000 cm^−1^ at a resolution of 4 cm^−1^.

### 2.5. Gas Chromatography-Mass Spectrometer

#### 2.5.1. Adsorption

About 3 g of each wood fiber sample was added into a 15 mL headspace bottle and conditioned at 60 °C in a water bath for 40 min; extraction occurred at the same temperature for 40 min using a 100 um PDMS extraction head (Supelco, Bellefonte, PA, USA). Desorption was carried out in the GC injection port.

#### 2.5.2. Chromatographic Conditions

GC separation was completed on an Innerwax 19091N-133 column (30 m × 0.25 mm; 0.25 μm, Agilent, Santa Clara, CA, USA) using helium as the carrier gas at a constant flow rate of 1.0 mL/min. The inlet temperature was set to 250 °C with a split column ratio of 50:1. The temperature of the column box was programmed to 40 °C, followed by an increase of 5 °C/min to 230 °C for at least 5 min.

#### 2.5.3. Mass Spectrometry

The GC-MS interface temperature was maintained at 280 °C, the ionization source temperature was maintained at 230 °C, and the quadrupole temperature was maintained at 150 °C. The electron impact ionization mode was used with a nominal electron energy (70 eV); the full scan range of samples for qualitative analysis ranged from 30 *m*/*z* to 550 *m*/*z*.

#### 2.5.4. Qualitative and Quantitative Analyses of Compounds

Compounds were identified by comparing the MS spectra to the NIST 2.0 library (National Institute of Standards and Technology, Gaithersburg, MD, USA). The relative content of each chemical component was calculated by area normalization and the average value of the three experiments.

Sample Availability: Samples of the compounds are available from the authors.

## 3. Results and Discussion

### 3.1. FTIR Analysis

**Figure 2:**
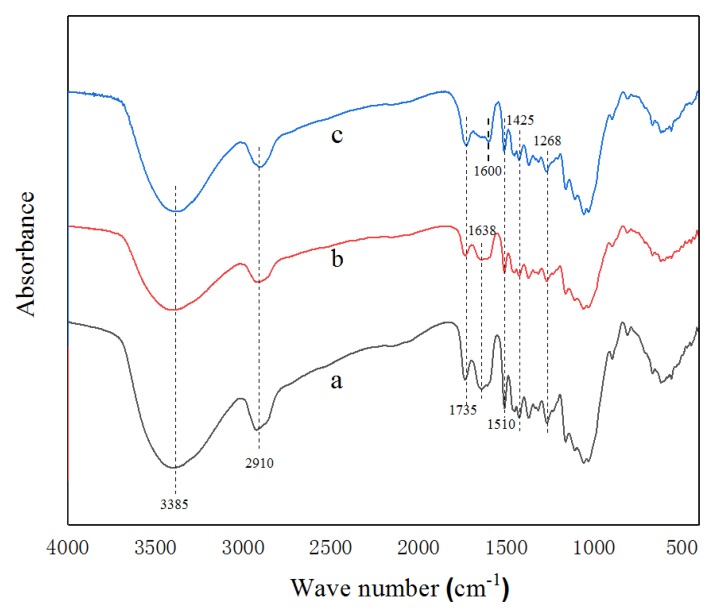
Fourier transform infrared spectroscopy (FTIR) spectra. (**a**) Untreated southern pine; (**b**) heat treated at 140 °C; (**c**) heat treated at 220 °C.

The FTIR spectra of untreated southern pine (heat treated at 140 °C and heat treated at 220 °C) are illustrated in Figure 2. Assignments of characteristic chemical bands from wood in the FTIR spectra were based on data from the literature. Detailed peak positions are summarized in Table 2.

Heat treatment has been found to induce chemical changes via deacetylation and release of acetic acid [19]. Dehydration of carbohydrates can reduce accessible OH groups and lead to the formation of furfural and hydroxymethylfurfural [20].

### 3.2. Identification of VOCs

**Figure 3:**
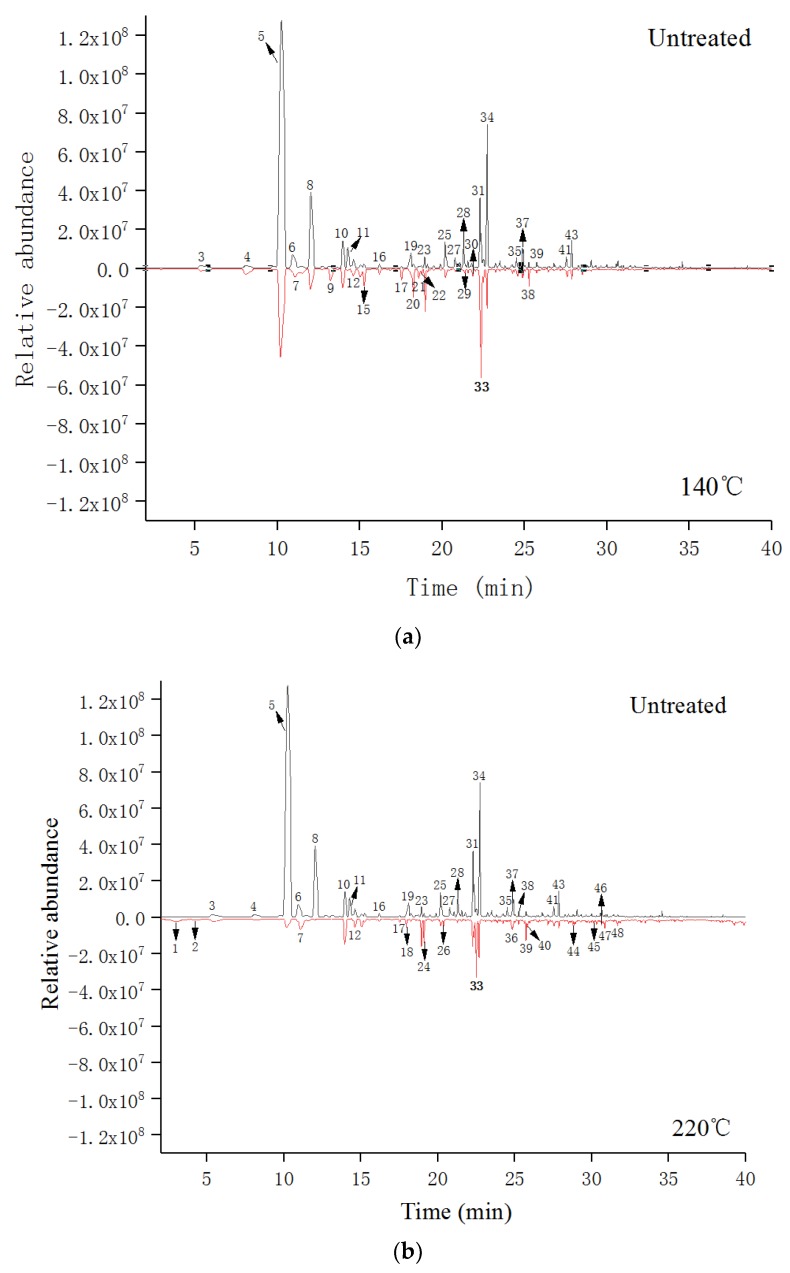
Gas chromatography-mass spectrometry (GC-MS) chromatograms of untreated southern pine and heat treated at 140 °C (**a**); untreated southern pine and heat treated at 220 °C (**b**). (peak numbers appear in Table 4).

The GC-MS chromatograms of untreated and heat-treated southern pine are presented in Figure 3. Results show a clear difference in peaks between untreated southern pine and that treated at 140 °C/220 °C in Figure 3a,b, respectively. The relative content of each component was obtained through area normalization, and the corresponding mass spectrum images of each chromatographic peak were searched using the National Institute of Standards and Technology (NIST) 2.0 standard library.

Results revealed 86 VOCs in untreated southern pine, and 93 VOCs were identified in southern pine after heat treatment at 140 °C. With an increase in heat-treatment temperature, the number of VOCs in southern pine increased to 131 at a temperature of 220 °C. In this case, the degradation of cellulose, hemicellulose, and lignin in southern pine at high temperatures could have formed new small molecular compounds [21], leading to growth in the number of VOCs in heat-treated southern pine. These compounds included alcohols, aromatics, acids, aldehydes, alkanes, alkenes, and some trace compounds (e.g., furans, ketones, phenols, and esters). In this paper, compounds with a peak area above 0.5% in southern pine were selected as key compounds, shown in Table 3.

Table 4 lists the key VOCs in southern pine before and after heat treatment. The contents of these compounds after heat treatment at different temperatures changed substantially. High-content (percentage > 5%) compounds included 2,6,6-trimethyl-bicyclo[3.1.1]hept-2-ene (peak 5, 49.19%), beta-pinene (peak 8, 9.61%), and (+)-alpha-terpineol(p-menth-1-en-8-ol) (peak 34, 6.83%) in the untreated southern pine (SP); 2,6,6-trimethyl-bicyclo[3.1.1]hept-2-ene is also called α-pinene, one of the main components of turpentine. As shown in Table 3, heat treatment can effectively reduce the contents of these compounds in untreated SP. When the temperature rose to 220 °C, the contents of α-pinene, beta-pinene, and (+)-alpha-terpineol(p-menth-1-en-8-ol) declined to 5.3%, 0%, and 2.07%, respectively.

**Figure 4:**
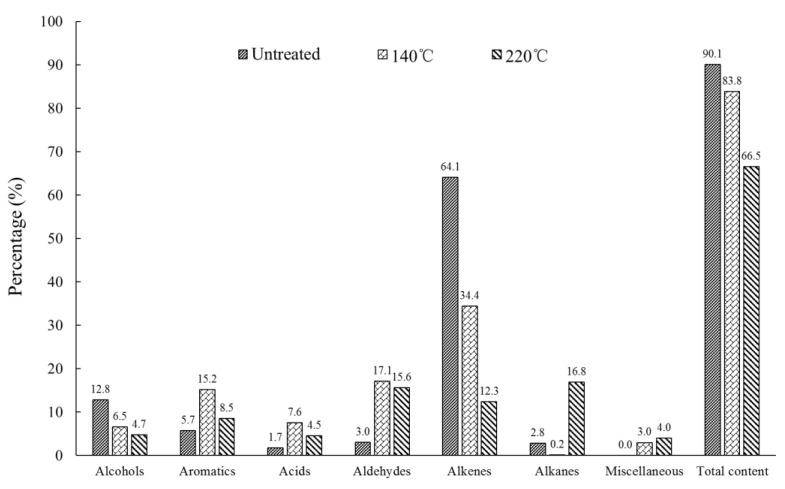
Contents of groups of volatile organic compounds in southern yellow pine before and after heat treatment at different temperatures.

The contents of VOC groups in untreated and heat-treated southern pine are presented in Figure 4. For alcohols, the group content reduced as the temperature increased. This pattern may have occurred because some polysaccharides in the hemicellulose of southern pine were cleaved to glucuronic aldehyde and some carbohydrates under high heat, resulting in decreased hydroxyl groups (including free hydroxyl groups) [22]. At the same time, a ‘bridge’ reaction occurred in each pair of free hydroxyl groups between cellulose molecular chains in the wood. Afterwards, an ether bond was formed by removing a water molecule; thus, the contents of the hydroxyl groups declined significantly [23].

The number of aromatics in untreated southern pine was low; however, with an increase in temperature, the content of aromatics increased considerably, presumably due to the degradation of lignin at high temperatures and the generation of aromatics [24]. 1-Methoxy-4-(2-propenyl)-benzene (peak 31, 4.87%), also called estragole and the main compound of aromatics in southern pine, reached boiling point at 216 °C. Therefore, when the temperature rose to 220 °C, the content of estragole reduced rapidly. 

High temperatures also increased the acid content in southern pine. The acid content was highest at 140 °C, presumably because the acetyl groups in hemicellulose degraded during heat treatment and generated acids [25]. However, as the temperature increased, the hydroxyl and carboxyl groups in southern pine reacted and formed esters and water [20]; therefore, when the temperature rose to 220 °C, the alcohol and acid contents in southern pine decreased, whereas that of the esters (1,3,3-Trimethyl bicyclo[2.2.1]hept-2-yl acetate, peak 32) increased.

Compared with untreated southern pine, many new aldehydes were identified in heat-treated southern pine; the degradation of lignin generated some aldehydes in the heat-treatment process [26]. 2-furancarboxaldehyde (peak 5) accounted for a large proportion of this group, and its content increased as the temperature rose. Inoue et al. found that hemicelluloses were degraded during heat treatment and could generate 2-furancarboxaldehyde and other substances [27], hence why the content of 2-furancarboxaldehyde increased at high temperatures.

Alkenes accounted for a large proportion of untreated southern pine. As the main compound of alkenes, 2,6,6-Trimethyl-bicyclo[3.1.1]hept-2-ene (peak 5, 49.19%), boiling point 156 °C, has been widely found in pine [28]. As the heat-treatment temperature increased, α-pinene released quickly; therefore, the alkene content fell from 64.1 to 12.3%. By contrast, the alkane content increased with increasing temperature, potentially due to pyrolysis of hemicelluloses.

Overall, compared to untreated southern yellow pine, at a temperature of 220 °C, alcohol and alkene contents were lowest and that of alkanes was highest. At a temperature of 140 °C, the contents of aromatics, acids, and aldehydes were highest. Excessive α-pinene, beta-pinene and (+)-alpha-terpineol (p-menth-1-en-8-ol) in untreated southern yellow pine pose threats to human health, yet heat treatment at 220 °C can effectively reduce the content of those compounds in southern yellow pine. Although heat treatment increased the amounts of VOCs, a high temperature can diminish the total contents of VOCs compared to untreated southern yellow pine.

## 4. Conclusions

Results revealed 86, 93, and 131 VOCs in untreated, 140 °C heat-treated, and 220 °C heat-treated southern yellow pine. These VOCs included alcohols, aromatics, acids, aldehydes, alkanes, alkenes, and some trace compounds (e.g., furans, ketones, phenols and esters). With an increase in heat-treatment temperature, the alkane content increased, whereas alcohol and alkene contents decreased. Contents of aromatics, acids, and aldehydes peaked at a heat-treatment temperature of 140 °C. In contrast to untreated southern yellow pine, heat treatment at 220 °C can effectively reduce the total contents of key VOCs in southern yellow pine but increase amounts of key VOCs. After heat treatment, the content of α-pinene, beta-pinene and (+)-alpha-terpineol(p-menth-1-en-8-ol) dropped significantly. Therefore, heat treatment in this paper can be used as a reference in the manufacturing process of wooden furniture or other indoor wooden materials made from southern yellow pine.

## Figures and Tables

**Table 1:** Essential parameters of southern yellow pine.

Label	SP
Wood	Southern yellow pine
Scientific name	*Pinus* spp.
Density (g cm^−3^)	0.49–0.53
Place of origin	Texas, USA
Water content (%)	8~12
Length	1500 mm
Condition of storage	10–40 °C, 30–70% RH, for one year

SP: southern yellow pine; RH: relative humidity.

**Table 2:** Main bands of infrared spectrum of wood and their assignment to functionality.

Wave Number	Functional Groups
3385	OH
2910	CH_3_-CH_2_-(cellulose)
1735	C=O (hemicellulose)
1638	C=C (conjugated carbonyl groups in lignin)
1600	A benzene ring skeleton (lignin)
1510	Aromatic ring skeleton retractable vibration
1425	C-H plane deformation (aromatic ring skeleton)
1268	C–O (guaiacyl ring in lignin)

**Table 3:** Key volatile organic compounds of southern yellow pine before and after heat treatment.

Group	Compounds
Alcohols	Ethanol; 1-Octanol; Exo-fenchol; (−)-Trans-pinocarveol; 4,6,6-Trimethylbicyclo[3.1.1]hept-3-en-2-ol; (+)-Alpha-terpineol(p-menth-1-en-8-ol); 1,7,7-Trimethyl-acetate-endo-bicyclo[2.2.1]heptan-2-ol
Aromatics	Benzene; Methyl(1-methylethyl)-benzene; P-cymene; Benzene; 1-Methoxy-4-(2-propenyl)-benzene
Acids	Acetic acid; Hexanoic acid; Cyclopentaneundecanoic acid; Heptanoic acid; (−)-(1s,2r,4r)-beta-fenchol; Octanoic acid; Nonanoic acid
Aldehydes	Hexanal; 2-Furancarboxaldehyde; Benzaldehyde; Octanal; (E)-2-octenal; Nonana; (Z)-6-nonenal; (Z)-2-decenal
Alkenes	2,6,6-Trimethyl-bicyclo[3.1.1]hept-2-ene;Beta-pinene;
	1-Methyl-4-(1-methylethenyl)-(s)-cyclohexene; 3-Isopropyl-6-methylene-1-cyclohexene;
	3-Methyl-6-(1-methylethylidene)-cyclohexene; Cetene;
	4,11,11-Trimethyl-8-methylene-[1r-(1r*,4e,9s*)]-bicyclo[7.2.0]undec-4-ene
Alkanes	2,2-Dimethyl-3-methylene-bicyclo[2.2.1]heptane;
	Dodecane; Tridecane; Pentadecane; 3-Methyl-tridecane; Heptadecane; Undecyl-cyclopentane; 2-Methyl-hexadecane; N-nonylcyclohexane; 3-Methyl-pentadecane; Hexadecane
Miscellaneous	2-Pentyl-furan; 2-Nonanone; 2-Methoxy-phenol; 1,3,3-Trimethylbicyclo[2.2.1]hept-2-yl acetate #

**Table 4:** Comparison of volatile organic compound (VOC) content in untreated southern pine (SP) and heat-treated SP.

Peak Number	RT (min)	Compounds	Percentage (%) *		
			Untreated	140 °C	220 °C
1	2.95	Ethanol	—	—	0.81
2	4.21	Benzene	—	—	0.76
3	5.37	Acetic acid	0.51	1.09	2.51
4	8.07	Hexanal	0.68	2.52	1.06
5	10.25	2,6,6-Trimethyl-bicyclo[3.1.1]hept-2-ene	49.19	24.37	5.3
6	10.93	2,2-Dimethyl-3-methylene-bicyclo[2.2.1]heptane	2.26	—	0.44
7	11.06	2-Furancarboxaldehyde	—	4.66	5.81
8	12.04	Beta-pinene	9.61	5.05	—
9	13.22	2-Pentyl-furan	—	2.4	0.17
10	13.97	1-Methyl-4-(1-methylethenyl)-(s)-cyclohexene	2.48	3.45	4.66
11	14.28	3-Isopropyl-6-methylene-1-cyclohexene	1.78	—	—
12	14.60	Methyl(1-methylethyl)-benzene	—	—	2.82
13	14.62	P-cymene	0.86	1.55	
14	15.05	Benzaldehyde	—	1.89	2.8
15	15.27	Octanal	—	2.45	0.77
16	16.19	3-Methyl-6-(1-methylethylidene)-cyclohexene	—	0.64	0.51
17	17.54	1-Methyl-4-(1-methylethenyl)-benzene	—	1.26	0.64
18	17.96	Hexanoic acid	—	—	1.18
19	18.20	(E)-2-octenal	1.65	—	0.32
20	18.26	Cyclopentaneundecanoic acid	—	4.02	—
21	18.57	1-Octanol	—	1.29	0.14
22	18.74	2-Nonanone	—	0.56	0.06
23	18.94	Nonanal	0.67	3.67	4.37
24	19.10	Dodecane	—	—	3.5
25	20.20	Exo-fenchol	1.81	1.23	0.88
26	20.37	2-Methoxy-phenol	—	—	0.98
27	20.78	(−)-Trans-pinocarveol	1.12	0.34	0.23
28	21.30	4,6,6-Trimethylbicyclo[3.1.1]hept-3-en-2-ol	1.62	—	—
29	21.38	Heptanoic acid	—	0.58	0.43
30	21.85	(Z)-6-nonenal	—	0.76	—
31	22.26	1-Methoxy-4-(2-propenyl)-benzene	4.87	12.39	4.27
32	22.34	1,3,3-Trimethylbicyclo[2.2.1]hept-2-yl acetate	—	—	2.8
33	22.50	Tridecane	—	—	5.26
34	22.74	(+)-Alpha-terpineol(p-menth-1-en-8-ol)	6.83	2.93	2.07
35	24.52	Octanoic acid	0.57	0.93	0.13
36	24.79	3-Methyl-tridecane	—	—	1
37	24.88	1,7,7-Trimethyl-acetate-endo-bicyclo[2.2.1]heptan-2-ol	1.44	0.74	0.6
38	25.27	(Z)-2-decenal	—	1.1	0.45
39	25.73	Heptadecane	0.50	0.15	2.36
40	25.80	Cetene	—	—	0.89
41	27.54	Nonanoic acid	0.62	0.95	0.28
42	27.54	Undecyl-cyclopentane	—	—	0.98
43	27.87	4,11,11-Trimethyl-8-methylene-[1r-(1r *,4e,9s *)]-bicyclo[7.2.0]undec-4-ene	1.03	0.86	0.98
44	28.78	Pentadecane	—	—	0.53
45	30.31	2-Methyl-hexadecane	—	—	0.6
46	30.64	N-nonylcyclohexane	—	—	0.67
47	30.85	3-Methyl-pentadecane	—	—	0.87
48	31.68	Hexadecane	—	—	0.63

* Percentage was calculated based on peak area. RT: retention time.
